# Supplementary material for: Quasi-experimental controlled study protocol to reduce sedentary lifestyle in patients with type 2 diabetes
Source: PLoS One. 2025 Sep 16;20(9):e0330393. doi: 10.1371/journal.pone.0330393 (PMC12440174; doi:10.1371/journal.pone.0330393)
Supplement: S2 Appendix — (PDF) [file pone.0330393.s002.pdf]

Anexo 2

MODELO PROTOCOLO ESTUDIO CUASI-EXPERIMENTAL

1. IDENTIFICACIÓN DEL PROTOCOLO

.. Número EUDRACT:_______________________
.. Código de protocolo del promotor:____________________
.. Versión/Fecha: Versión 2.0, 11/09/2024

2. TÍTULO DEL ESTUDIO

Análisis de la efectividad de una intervención basada en la disminución del comportamiento sedentario en población con Diabetes Mellitus tipo 2.

3. IDENTIFICACIÓN DEL PROMOTOR

No procede.

4. DATOS ECONÓMICOS Y ASPECTOS RELEVANTES SOBRE LA FINANCIACIÓN DEL ESTUDIO

Los datos económicos del estudio se detallan a continuación.

1. **Instalaciones**

   Ubicación. Asociación de Diabéticos de La Rioja (ASDIR).
   Coste. Uso gratuito de las instalaciones propias de la asociación.

2. **Equipamiento y material**

   Acelerómetros (Actigraph GT3X BT):
   Proveedor. Universidad de Zaragoza.
   Cantidad. 19 unidades.
   Coste. Préstamo sin coste.

3. **Servicios médicos**

   Analíticas y pruebas complementarias:
   Realización. Centros de salud de correspondencia.
   Coste. Servicios ofrecidos como parte de la atención sanitaria regular, sin coste adicional para el estudio.

4. **Personal**

   Investigadores y Colaboradores. El estudio será realizado por los investigadores participantes de forma voluntaria.
   Coste. Sin inversión en personal remunerado.

5. **Infraestructuras**

   Infraestructuras adicionales. No se requiere inversión en infraestructuras adicionales.
   Coste. Sin coste.

6. **Otros gastos.**

   Gastos Admimnistrativos. No se prevén gastros administrativos adicionales.
   Coste. Sin coste.

7. **Gastos de Difusión y Publicación de resultados.**

   Difusión y Publicación de resultados. Publicación en revistas científicas y asistencia a Congresos Sanitarios.
   Coste de Publicación en Revistas. 1.500 – 5.000 euros.
   Coste de Inscripción a Congresos. 200 – 1.000 euros.

El estudio cuenta con recursos limitados y no recibirá financiación de ninguna fuente externa. El trabajo relacionado con la concepción y diseño e informe del estudio ha sido realizado sin apoyo financiero externo. Como se ha detallado, los acelerómetros utilizados en este estudio han sido prestados por la Universidad de Zaragoza y, aparte de esta provisión de equipo, no ha habido otras formas de apoyo material.

Actualmente, los costes asociados a la publicación en revistas e inscripción a congresos no están cubiertos por ninguna institución. Sin embargo, se considera la posibilidad de solicitar becas o subenciones en un futuro para cubrir estos gastos.

5. INVESTIGADOR COORDINADOR DEL ESTUDIO EN ESPAÑA.

Elena Andrade Gómez.

DIRECCIÓN DE SU CENTRO DE TRABAJO. Unidad Predepartamental de Enfermería, Universidad de La Rioja.

6. CEIC DE REFERENCIA

Para la revisión y aprobación de este estudio, se ha elegido el Comité Ético de Investigación con medicamentos de La Rioja (CEImLar) por varias razones. En primer lugar, su localización geográfica, pues el CEImLar está localizado en proximidad al lugar donde se llevará a cabo el estudio, lo que facilita la comunicación y gestión de documentos. Adicionalmente, su especialización y experiencia en estudios de nuestra área terapéutica garantiza una evaluación precisa y pertinente. El CEImLar también cuenta con la acreditación necesaria para la revisión de estudios clínicos de nuestra región. Estas razones aseguran que el CEImLar es el más adecuado para la revisión ética de nuestro estudio.

7. CENTROS DONDE SE PREVÉ REALIZAR EL ESTUDIO (ver Anexo 1)

Las sesiones grupales presenciales y la visita individual presencial con el grupo de intervención serán llevadas a cabo en la ASDIR. Las mediciones del estudio se realizarán en los centros de salud de correspondencia, en las cuales se incluye la medición de la tensión arterial, mediciones antropométricas y extracciones analíticas. La colocación de los acelerómetros se realizará en la ASDIR por la enfermera encargada del estudio.

8. JUSTIFICACIÓN Y PERTINENCIA DEL ESTUDIO

La prevalencia del sedentarismo en Europa es muy elevada, y en España ha ido en aumento considerable en las últimas décadas (1). Según los datos recogidos por el Instituto Nacional de Estadística (INE), en 2020 el porcentaje de sedentarismo entre adultos españoles en edad de 25 a 30 años superó el 30% y se aproximó al 50% en adultos en edad superior a 75 años (2). La Organización Mundial de la Salud (OMS) en un documento del año 2004 actualizado en 2010, clasificó el sedentarismo como el cuarto factor de riesgo de mortalidad mundial (3).

El comportamiento sedentario está sujeto al aumento de los factores de riesgo independientemente de los niveles de actividad física (4,5). Varios estudios observacionales respaldan un riesgo de mortalidad superior por todas las causas para las personas con un mayor nivel de sedentarismo (6–8). Adicionalmente, los efectos beneficiosos de la actividad física podrían verse disminuidos por un exceso del tiempo sedentario (9–11).

Estilos de vida sedentarios conllevan al incremento de la prevalencia de alteraciones funcionales y orgánicas como obesidad, síndrome metabólico, disminución de la sensibilidad a la insulina, diabetes mellitus tipo 2, enfermedades cardiovasculares y al padecimiento de distintos tipos de cánceres (4,12–18).

El interés en la conducta sedentaria está justificado por el incremento del tiempo de sedentarismo en la población, así como por las enfermedades derivadas de la inactividad física. Considerándose necesario, además del incremento de la actividad física, la implementación de estrategias que reduzcan el comportamiento

sedentario. Este puede tratarse de un objetivo más agible como estrategia suplementaria para la mejora de la condición física.

Por otro lado, la diabetes tipo 2 es una enfermedad crónica no transmisible con gran impacto en la salud de la población a nivel mundial, siendo la causa de más de 4 millones de muertes cada año (19,20). Las proyecciones de la Federación Internacional de la Diabetes (FID), señalan que en 2045, 1 de cada 8 personas o lo que supone aproximadamente 783 millones de personas vivirán con diabetes, esto conjetura un aumento del 46% con respecto a 2021 (20).

La prevalencia de esta enfermedad ha aumentado considerablemente en adolescentes y en adultos jóvenes. Esto es debido a cambios en la alimentación, aumento del consumo de bebidas carbonatadas, disminución de la actividad física, consumo de alcohol, hábito tabáquico, así como el sedentarismo, todos ellos factores potencialmente modificables (21,22). Adicionalmente, la situación vivida por la pandemia por SARS-CoV-2 aumentó el número de casos de personas diabéticas y los factores de riesgo de padecer diabetes (21).

Está ampliamente demostrado que el sedentarismo está estrechamente vinculado a niveles superiores de glucosa e insulina postprandial, lo que propende a un aumento de peso, desarrollo de resistencia insulínica y a altos niveles de adipocinas, originando un estado proinflamatorio crónico (23,24). Además, se conoce que un mayor número de horas tumbado es el principal causante de la inflexibilidad metabólica, que además de los efectos anteriormente expuestos, conllevan a cambios en el tipo de fibra muscular y al almacenamiento de grasa ectópica (hígado y corazón grasos) (25–28).

En general, el sedentarismo y la diabetes tienen consecuencias notablemente negativas para la salud, pero no únicamente a nivel físico sino también mental, pudiendo desencadenar problemas psicosociales, lo que obstaculiza el autocuidado (29). Ambos, son comportamientos integrados en la conducta, y es por ello por lo que pueden ser asumidos como costumbres en el estilo de vida de las personas (30). Para intervenir en estos estilos de vida se debe tratar la conducta para la creación de hábitos que supongan un beneficio en la calidad de vida. Y, para programar una intervención eficaz, es necesario tener en cuenta la adherencia, ya que de ello dependerá la consecución de los resultados. Cómo conseguir un nivel de adherencia alto es uno de los principales retos en los programas de intervención, para ello, la motivación es el factor clave, y será lo que en última estancia haya conllevado al establecimiento de un nuevo hábito (31).

Nuestro estudio evalúa el comportamiento sedentario de los pacientes tanto cualitativa como cuantitativamente. Los esfuerzos para disminuir el sedentarismo son muy necesarios y son pocos los estudios que han evaluado la relación entre el sedentarismo y la diabetes de forma independiente de la cantidad de ejercicio físico realizado, por ello nuestra intervención no está enfocada en aumentar el ejercicio físico sino en reducir el tiempo de sedentarismo. Por último, si se verifica que nuestra intervención tiene efectos favorables y mantenidos en el tiempo, orientará a la elaboración de recomendaciones clínicas y de salud pública.

HIPÓTESIS:

La intervención basada en visitas grupales, individuales e intervención telefónica personalizada dirigida por enfermeras conllevará a modificaciones en el estilo de vida (reducción del tiempo de sedentarismo), en parámetros antropométricos y parámetros bioquímicos a los 6 meses de seguimiento. Además, los hábitos adquiridos, serán mantenidos durante los 6 meses posteriores al periodo de intervención.

9. DISEÑO

Se trata de un estudio cuasi-experimental de 12 meses de seguimiento. Se ha optado por llevar a cabo el estudio como unicéntrico por razones estratégicas y logísticas. Este tipo de estudio permite una gestión más eficiente de los recursos, además la implementación uniforme de los protocolos en un centro garantiza una mayor consistencia en la recopilación de datos y permite una gestión más eficiente de los recursos. Adicionalmente, el centro elegido tiene una notable experiencia y especialización en nuestra área terapéutica, pues la Asociación de Diabéticos de La Rioja (ASDIR) es una organización dinámica con más de 40 años de experiencia, compuesta

por personas de todas las edades que tienen diabetes, así como de sus familiares, allegados, voluntarios y colaboradores.

El presente estudio utilizará un diseño de grupos paralelos. Un grupo recibirá una intervención de seis meses de duración en la que se incluirán dos sesiones grupales, llamadas telefónicas bimestrales y una visita presencial con la enfermera encargada del estudio, el grupo control únicamente recibirá cada dos meses información detallada por escrito sobre hábitos de vida saludables por correo electrónico. Esto permite comparar directamente los efectos de la intervención frente al grupo que no la recibe, asegurando que los resultados sean atribuibles a las intervenciones específicas administradas a cada grupo.

Con respecto a la asignación y enmascaramiento, la distribución en el grupo control o grupo de intervención será realizada de manera secuencial en proporción 1:1. La estratificación será realizada por orden de acceso al estudio, el primero de los pacientes que acepte el consentimiento informado se incluirá en el grupo intervención, el segundo en el grupo control y así sucesivamente hasta completar la muestra. Debido a la naturaleza de la intervención, los participantes, la enfermera y el personal que colabore conocerán los grupos asignados, no obstante, estará cegado para los evaluadores de resultados.

10. OBJETIVO PRINCIPAL

El objetivo principal del estudio es examinar, a través de un estudio cuasi-experimental, el efecto de la disminución del tiempo de sedentarismo y el aumento de la motivación para adoptar un estilo de vida activo sobre diferentes parámetros clínicos, antropométricos y bioquímicos relacionados con la salud en pacientes diagnosticados con diabetes mellitus tipo 2.

Los objetivos generales son:

Objetivo 1. Evaluar la calidad de vida y el grado de motivación de los pacientes tras intervenciones dirigidas a la disminución del tiempo de sedentarismo.

Objetivo 2. Integrar en el estilo de vida de los participantes la disminución del tiempo de sedentarismo con herramientas y orientaciones prácticas adaptadas a las circunstancias.

Objetivo 3. Implementar un formulario de registro de la valoración enfermera que posibilite la evaluación del comportamiento sedentario de la población en la plataforma Selene del Servicio Riojano de Salud.

11. FÁRMACO EXPERIMENTAL Y CONTROL. DOSIS, FORMA FÍSICA, VÍA DE ADMINISTRACIÓN, GRUPO TERAPÉUTICO

No pocede.

12. VARIABLE PRINCIPAL DE VALORACIÓN

Las variables de resultado principales del estudio son el nivel de actividad y estado de motivación al cambio, para ello, se diseñará un cuestionario estructurado en varios apartados con ítems extraídos de las versiones españolas validadas del Cuestionario Internacional de Actividad Física (IPAQ), Cuestionario de Comportamiento Sedentario (SBQ-s), Cuestionario de Regulación Conductual en el Ejercicio Físico (BREQ-3) y Cuestionario de la Calidad de Vida en Personas con Diabetes (DQOL). Adicionalmente, el tiempo de sedentarismo se evaluará mediante el uso de acelerómetros.

Las variables de resultado secundarias son el peso (Kg), talla (cm), IMC (peso en Kg/altura en m2), perímetro de cintura y cadera (cm), biomarcadores sanguíneos (glucosa, hemoglobina glucosilada, insulina, Homa-IR, colesterol total, colesterol HDL, colesterol LDL, triglicéridos, índice de aterogénico, proteína C reactiva, vitamina D e Interleucina 6 (IL-6)), la tensión arterial, así como datos sociodemográficos, laborales y clínicos que serán recogidos mediante hojas de registro diseñadas.

La variable independiente principal será el grupo de pertenencia (grupo de intervención o grupo control).

13. POBLACIÓN EN ESTUDIO Y NÚMERO TOTAL DE PACIENTES

Se ha calculado el tamaño muestral en relación con 230 personas con diabetes mellitus tipo 2 asociadas a la ASDIR. Se ha utilizado un nivel de confianza del 95% y un margen de error del 5%, posteriormente se ha ajustado el tamaño de la muestra anticipando una tasa de pérdidas del 15%, por tanto el tamaño final de la muestra ajustado es de 169 personas.

Para el procedimiento de selección y reclutamiento se estableció contacto con la ASDIR para conocer la asociación y describir el proyecto. Para darlo a conocer a los asociados y conocer su disposición para participar, el presidente de la asociación procederá al envío de correos electrónicos y un mensaje de difusión en WhatsApp por el grupo de la comunidad. Las personas interesadas y que cumplan con los criterios de inclusión, serán seleccionadas para el estudio. Se realizará una visita presencial en la Asociación para explicar las bases del proyecto detalladas y para la firma del consentimiento informado.

Los criterios de inclusión aplicados son:

- Diagnóstico de diabetes mellitus tipo 2 y cumplimiento de uno de los siguientes criterios: que en dos análisis el valor de hemoglobina glucosilada (HbA1C) sea mayor o igual a 6,5% o que al menos en dos análisis los niveles de glucosa plasmática en ayunas sean superiores o iguales a 126 mg/dL.
- Edad igual o superior a 18 años.
- Aptitud física mínima.

Los criterios de exclusión son:

- Contraindicación médica.
- Mujeres embarazadas.
- Intervenciones quirúrgicas recientes que limiten la movilidad.
- Pacientes en tratamiento oncológico en la actualidad o menor de un año desde el fin del tratamiento.

Aquellos participantes interesados firmarán el consentimiento informado una vez verificados los criterios de elegibilidad.

14. ANÁLISIS ESTADÍSTICO

Para realizar el análisis estadístico se compararán el grupo de intervención y el grupo control. Para la elaboración de los resultados se utilizará *Microsoft Excel* versión 2022 y para la realización del análisis estadístico la plataforma *IBM SPSS Statistics*.

Las variables cuantitativas serán señaladas como medias ± desviación estándar y cuando no sean normales, se presentarán con la mediana (percentiles 25 y 75). Las variables cualitativas se describirán como frecuencias relativas, frecuencias relativas acumuladas y porcentajes. La significancia estadística se establecerá para valores de p menores de 0,05 ($p<0,05$). Y, para comparar si hay significación estadística entre ambos grupos se aplicará la prueba chi cuadrado.

15. CONSIDERACIONES ÉTICAS

El presente estudio cumplirá con todas las normas éticas establecidas. Los profesionales sanitarios que participen en el estudio firmarán un documento en el que se comprometen a proteger la confidencialidad de todos los pacientes. Antes de realizar cualquier procedimiento, se deberá obtener el consentimiento informado de cada paciente, que podrá ser revocado voluntariamente en cualquier momento del estudio.
Se seguirán las directrices establecidas en la Declaración de Helsinki, las normas de buena práctica que rigen cualquier estudio clínico y la Ley de protección de datos y la legislación vigente en España. El tratamiento de los datos de carácter personal de todos los pacientes se ajustará a lo dispuesto en la Ley Orgánica 3/2018, de 5 de

diciembre, de Protección de Datos Personales y garantía de los derechos digitales y a lo estipulado en el Reglamento (UE) 2016/679 del Parlamento europeo y del Consejo de 27 de abril de 2016 de Protección de Datos (RGPD).

Con respecto a los riesgos asociados al estudio, los acelerómetros de cinta usados en la cintura para monitorizar la actividad pueden resultar incómodos para algunos participantes, no obstante, la intervención no implica ningún otro riesgo físico. Entre los riesgos psicológicos se incluyen estrés, ansiedad o cambios en el comportamiento secundarios a la modificación de la rutina diaria o de los hábitos de vida. Los beneficios de participar en el estudio son la mejora de la salud física, mejora del control glucémico, disminución del riesgo de enfermedades cardiovasculares u obesidad, mejora del estado de ánimo y aumento de la energía y vitalidad entre otros. De manera que los beneficios de participar en el estudio para reducir el sedentarismo son numerosos y significativos para la salud física y mental. Los riesgos son principalmente psicológicos y temporales, puesto que no se exige la realización de ejercicio físico intenso, sino la disminución del tiempo en actividades sedentarias. Por otra parte, destacar que no existen compensaciones económicas por la participación en el estudio.

La elección de las intervenciones, que incluyen llamadas telefónicas y visitas presenciales tanto grupales como individuales radican de la necesidad de intervenciones educativas. Entre los métodos más utilizados y evaluados para mejorar la adherencia al tratamiento se encuentran las intervenciones educativas y las llamadas telefónicas. Estas intervenciones han demostrado ser efectivas para mejorar la adherencia al tratamiento en pacientes con diversas patologías. Numerosos estudios evidencian que los pacientes tienden a ser más honestos en las entrevistas telefónicas que en las entevistas cara a cara con los profesionales. Además, son un método de bajo coste económico y requieren menos tiempo y personal, convirtiéndose en una herramienta con gran potencial para implementar en los centros de salud, especialmente en contextos de escasez de personal y aumento de la demanda. Asimismo, la intervención educativa grupal es una estrategia comunmente utilizada que ha demostrado resultados muy favorables en cuanto a adquisición de conocimientos, los cuales persisten en el tiempo con apoyo de una educación individual continua. Para el grupo control, se establecerán únicamente mensajes sobre hábitos de vida saludables por correo electrónico, incidiendo en la importancia de mantener un estilo de vida activo y en los riesgos asociados al sedentarismo, también se les proporcionará material educativo estándar sobre el manejo de la diabetes mellitus tipo 2. Desde el punto de vista ético, resulta aceptable ya que los participantes deberán estar plenamente informados sobre la estructura del estudio y proporcionarán su consentimiento libre y voluntario, además, deberán saber que forman parte de un grupo control y entender lo que ello implica. Si la intervención demuestra ser beneficiosa, se puede considerar su ofrecimiento al grupo control una vez que el estudio haya concluido. El diseño de nuestro estudio respeta los principios éticos fundamentales y protege el bienestar y los derechos de todos los participantes.

La información se dará por escrito a los sujetos participantes. En los casos en los que los participantes no puedan tomar decisiones por sí mismos, la información será proporcionada a los familiares o representantes legales según proceda.

Se permitirán las monitorizaciones, auditorias, revisiones del CEImLar e inspecciones reguladoras con el estudio, facilitando el acceso directo a los datos originales.

16. DURACIÓN DEL TRATAMIENTO

La difusión del proyecto tendrá un periodo total de dos meses, la selección de los participantes se realizará en el mes posterior y la firma del consentimiento informado se alargará hasta el mes siguiente, paralelamente se realizará la asignación de los grupos. El estudio tendrá una duración de 12 meses completos a partir de la fecha de firma del consentimiento informado. Se realizará una valoración inicial a ambos grupos al inicio del estudio, las mediciones posteriores se realizarán a los 3, 6 y 12 meses. El periodo de intervención comprende los 6

primeros meses, en el cual el grupo control recibirá cada dos meses información detallada de los hábitos de vida saludables a través del correo electrónico, es decir, en el segundo, cuarto y sexto mes, a partir de entonces, este grupo no recibirá atenciones adicionales durante el periodo del estudio. Para el grupo de intervención se realizarán dos sesiones grupales presenciales, la primera en el primer mes y la segunda en el quinto, también se llevará a cabo una visita individual de carácter presencial en el tercer mes y se establecerá contacto telefónico cada dos meses, esto es en el segundo, cuarto y sexto mes. Una vez transcurridos los 12 meses se dará por finalizado el estudio, ello implica la no realización de intervenciones adicionales ni seguimientos para cualquiera de los participantes del estudio.

17. EVALUACIÓN DE LA SEGURIDAD DEL ESTUDIO

No procede.

18. CALENDARIO Y FECHA PREVISTA DE FINALIZACIÓN

Ver cronograma en última página del anexo.

REFERENCIAS

1. Montero-Torreiro MF, Rey-Brandariz JR, Guerra-Tort C, Candal-Pedreira C, Santiago-Pérez MI, Varela-Lema L, Suárez S P-RM. Evolución de la prevalencia de sedentarismo en la población española entre los años 1987 y 2020. Med Clin (Barc). 2024;162(6):273–9.
2. Sedentarismo según grupos de edad y periodo. Instituto Nacional de Estadística [Internet]. 2022. Available from: https://ine.es/jaxi/Datos.htm?path=/t00/mujeres_hombres/tablas_1/l0/&file=d06006.px
3. Global Health Risks: Mortality and burden of disease attributable to selected major risks. [Internet]. World Health Organization. 2009. Available from: http://www.who.int/healthinfo/global_burden_disease/GlobalHealthRisks_report_full.pdf
4. Leiva AM, Martinez MA, Cristi-Montero C, Salas C, Ramírez-Campillo R, Díaz Martínez X, Aguilar-Farias N C-MC. Sedentary lifestyle is associated with metabolic and cardiovascular risk factors independent of physical activity. Rev méd Chile. 2017;145(4):458–67.
5. Biswas A, Oh PI, Faulkner GE, Bajaj R, Silver M, Mitchell M AD. Sedentary time and its association with risk for disease incidence, mortality, and hospitalization in adults: a systematic review and meta-analysis. Ann Intern Med. 2015;162(2):123–32.
6. Bankoski A, Harris T, McClain J, Brychta R, Caserotti P, Chen K, Berrigan D, Troiano R KA. Sedentary activity associated with metabolic syndrome independent of physical activity. Diabetes Care. 2011;34(2):497–503.
7. Koster A, Caserotti P, Patel K, Matthews C, Berrigan D, Van Domelen D, Brychta R, Chen K HT. Association of sedentary time with mortality independent of moderate to vigorous physical activity. PLoS One. 2012;7(6):e37696.
8. Chomistek A, Manson J, Stefanick M, Lu B, Sands-Lincoln MS. Going S, Garcia L, Allison M, Sims S, LaMonte M, Johnson C EC. Relationship of sedentary behavior and physical activity to incident cardiovascular disease: results from the Women's Health Initiative. J Am Coll Cardiol. 2013;61(23):2346–54.
9. Cristi-Montero C, Celis-Morales C, Ramírez-Campillo R, Aguilar-Farías N, Álvarez C R-RF. Sedentary behaviour and physical inactivity is not the same!: An update of concepts oriented towards the prescription of physical exercise for health. Rev Med Chil. 2015;143(8):1089–90.
10. Cristi-Montero C. Considerations regarding the use of metabolic equivalents when prescribing exercise for health: preventive medicine in practice. Phys Sport. 2016;44(2):109–11.
11. Kulinski J, Khera A, Ayers C, Das S, A de Lemos J, Blair S BJ. Association between cardiorespiratory fitness and accelerometer-derived physical activity and sedentary time in the general population. Mayo Clin

Proc. 2014;89(8):1063–71.

12. Lee IM, Shiroma EJ, Lobelo F, Puska P, Blair SN, Katzmarzyk PT LPASWG. Impact of physical inactivity on the world´s major non-communicable diseases. Lancet. 2012;380:219–29.
13. Rynders C, Blanc S, DeJong N, Bessesen D BA. Sedentary behaviour is a key determinant of metabolic inflexibility. J Physiol Pathophysiol. 2018;1319–30.
14. Ryan D, Stebbings G OG. The emergence of sedentary behaviour physiology and its effects on the cardiometabolic profile in young and older adults. Age (Omaha). 2015;37(89).
15. Young D, Hivert MF, Alhassan S, Camhi S, Ferguson J, Katzmarzyk P, Lewis C, Owen N, Perry C, Siddique J YC. Sedentary Behavior and Cardiovascular Morbidity and Mortality: A Science Advisory From the American Heart Association. Am Hear Assoc. 2016;13:262–79.
16. Nomura S, Dash C, Sheppard V, Bowen D, Allison M, Barrington W, Chlebowski R, Coday M, Hou L, Howard B, LaMonte , Manson J, Neuhouser M, Paskett E, Sattari M, Stefanick M, Wactawski-Wende J A-CL. Sedentary time and postmenopausal breast cancer incidence. Cancer causes Control. 2017;28:1405–16.
17. Zhou Y, Zhao H PC. No TitleAssociation of sedentary behavior with the risk of breast cancer in women: update meta-analysis of observational studies. Ann Epidemiol. 2015;25(9):687–97.
18. Cong Y, Gan Y, Sun H, Deng J, Cao S, Xu X LZ. Association of sedentary behaviour with colon and rectal cancer: a meta-analysis of observational studies. Br J Cancer. 2014;110(3):817–26.
19. Blanco E, Chavarría F GY. Healthy lifestyle in type 2 diabetes mellitus: benefits in chronic management. Rev Médica Sinerg. 2020;6(2):639.
20. Datos y cifras sobre la diabetes. Int Diabetes Fed [Internet]. 2021; Available from: https://idf.org/es/about-diabetes/diabetes-facts-figures/
21. Acosta L, Angarita M OL. Diabetes mellitus tipo 2: Latinoamérica y Colombia, análisis del último quinquenio. Rev Med. 2023;31(2):35–46.
22. Leiva A, Martínez M, Pettermann F, Garrido-Méndez A, Poblete-Valderrama F, Díaz-Martínez X C-MC. Risk factors associated with type 2 diabetes in Chile. Nutr Hosp. 2018;35:400–7.
23. Blancas-Flores G, Almanza-Pérez J, Lopez-Roa R, Alarcon F G-MM. La obesidad como un proceso inflamatorio. Bol Med Hosp Infant Mex. 2010;67(2):88–97.
24. Martinez-Ferran M, Guía-Galipienso F, Sanchis-Gomar F P-GH. Metabolic Impacts of Confinement during the COVID-19 Pandemic Due to Modified Diet and Physical Activity Habits. Nutrients. 2020;12(6):1549.
25. Bergouignan A, Rudwill F, Simon C BS. Physical inactivity as the culprit of metabolic inflexibility: Evidences from bed-rest studies. J Appl Physiol. 2011;111(4):1201–10.
26. Alibegovic A, Højbjerre L, Sonne M, Van G, Stallknecht B, Dela F VA. Impact of 9 days of bed rest on hepatic and peripheral insulin action, insulin secretion, and whole-body lipolysis in healthy young male offspring of patients with type 2 diabetes. Diabetes. 2009;58(12):2749–56.
27. Cellini N, Canale N, Mioni G CS. Changes in sleep pattern, sense of time and digital media use during COVID-19 lockdown in Italy. J Sleep Res. 2020;29(4):13073.
28. Narici M, De Vito G, Franchi M, Paoli A, Moro T, Marcolin G, Grassi B, Baldassarre G, Zuccarellli L, Biolo G, Girolamo F, Fiotti N, Dela F, Greenhaff P MC. Impact of sedentarism due to the COVID-19 home confinement on neuromuscular, cardiovascular and metabolic health: Physiological and pathophysiological implications and recommendations for physical and nutritional countermeasures. Eur J Sport Sci. 2021;21(4):614–35.
29. ADA. Facilitating Behavior Change and Well-being to Improve Health Outcomes: Standards of Medical Care in Diabetes-2022. Diabetes Care [Internet]. 2022;1(45):60–82. Available from: https://pubmed.ncbi.nlm.nih.gov/34964866/
30. Ríos D. Epidemiología de la actividad física en la Unión Europea niveles de actividad, lesiones deportivas y motivaciones. Digitum. 2013.

31. Corral JA. Actividad física, estilos de vida y adherencia de la práctica de actividad física de la población adulta de Sevilla. 2016.

ANEXO I:

**Investigadora principal**	**Centro**
Raquel Sainz Prado	Universidad de La Rioja

Tabla 1. Cronograma del estudio.

(2024-2025)											
	SEPT.	OCT.	NOV.	DIC.	ENE.	FEB.	MAR.	ABR.	MAY.	JUN.	JUL.
Presentación del Proyecto a CEImLar											
Presentación del Proyecto a la Asociación de Diabetes de La Rioja											
Difusión del proyecto a los asociados											
Selección de los participantes											
Firma consentimiento informado											
Asignación de los grupos											
Recogida de datos inicial											
Periodo del estudio											
Periodo de intervención											
Difusión de mensajes por correo en el grupo control											
Sesiones grupales con el grupo de intervención											

**(2024-2025)**											
	SEPT.	OCT.	NOV.	DIC.	ENE.	FEB.	MAR.	ABR.	MAY.	JUN.	JUL.
Llamadas telefónicas al grupo de intervención							■		■		■
Visita individual presencial con el grupo de intervención								■			
Mediciones						■		■			■
**(2025-2026)**											
	SEPT.	OCT.	NOV.	DIC.	ENE.	FEB.	MAR.	ABR.	MAY.	JUN.	JUL.
Periodo del estudio	■	■	■	■	■	■					
Mediciones						■					
Análisis de datos							■	■	■		
Redacción del Informe final										■	■
