# Supplementary material for: Quasi-experimental controlled study protocol to reduce sedentary lifestyle in patients with type 2 diabetes
Source: PLoS One. 2025 Sep 16;20(9):e0330393. doi: 10.1371/journal.pone.0330393 (PMC12440174; doi:10.1371/journal.pone.0330393)
Supplement: S3 Appendix — (DOCX) [file pone.0330393.s003.docx]

**PERSONAL DATA**

| First name and second name. | |
| --- | --- |
| Date of birth. | Age. |
| Telephone number. | E-mail. |
| Place of birth. | Correspondence Health center. |
| Pace of residence. |  |

**MEDICAL HISTORY**

**Do you suffer from any chronic disease? If you suffer from any of the above, mar kit with an X or specify which disease it is.**

| Hypertension |  |
| --- | --- |
| Diabetes, since when? |  |
| Hypercholesterolemia |  |
| Chronic obstructive pulmonary disease |  |
| Heart failure or ischemic heart disease |  |
| Peripheral arterial disease |  |
| Chronic renal insufficiency |  |
| Deppression |  |
| Cancer |  |
| Arthritis |  |
| Osteoarthritis |  |
| Osteoporosis |  |
| Other |  |

**Medication**

| What medications do you take on a regular basis? Indicate which ones and for what purpose. |
| --- |
| Do you take any medication on an occasional basis? Indicate which ones and what for. |

**Personal history of diabetes**

| Do you have any relatives who suffer or have suffered from type 2 diabetes mellitus? If yes, please specify relationship. |
| --- |

**Frequency of Health care**

| In relation to diabetes, how many times in a three-month period do you go to your primary care physician? |
| --- |

**Tobacco use (Indicate answer with X)**

| **Yes** |  |
| --- | --- |
| Number of cigarettes per day |  |
| Number of years smoking |  |
| **Ex–smoker** (not smoked in the last 6 months) |  |
| **No** |  |

**Alcohol consumption (Indicate answer with X)**

| **Yes** |  |
| --- | --- |
| Daily (indicate number of drinks per day) |  |
| Weekend (indicate number of total weekend drinks) |  |
| **No** |  |

**SOCIODEMOGRAPHIC DATA**

**Educational level (Indicate answer with X)**

| No education |  |
| --- | --- |
| Primary education |  |
| Secondary education |  |
| Certificate of professionalism |  |
| Vocational training |  |
| University studies |  |

**Marital status (Indicate answer with X)**

| Single |  |
| --- | --- |
| Married |  |
| Domestic partnership |  |
| Divorced |  |
| Separated |  |
| Widowed |  |

**Work activity (Indicate the answer with X)**

| Active |  |
| --- | --- |
| Pre retired |  |
| Retired |  |
| Occupation |  |
| For how many years have you been in the occupation |  |

**SPORTS RECORD**

| In the past, have you continuously practiced any sport? | Indicate which |
| --- | --- |
| For how long (years)? |  |
| What is the highest level of sport in which you have participated? |  |

**Injuries**

| Have you suffered any major sport or physical activity-related injury in the past? | Indicate which and the year |
| --- | --- |
| Have you had any surgical intervention derived from an injury or problems related to the practice of sports in the past? | Indicate which and the year |
| In the past, have you had any major surgery or injury NOT related to sports? | Indicate which and the year |
