# Supplementary material for: Quasi-experimental controlled study protocol to reduce sedentary lifestyle in patients with type 2 diabetes
Source: PLoS One. 2025 Sep 16;20(9):e0330393. doi: 10.1371/journal.pone.0330393 (PMC12440174; doi:10.1371/journal.pone.0330393)
Supplement: S4 Appendix — (DOCX) [file pone.0330393.s004.docx]

**APPENDIX 4. SEDENTARY BEHAVIOR QUESTIONNAIRE (SBQ)**

| **SEDENTARY BEHAVIOR: Weekday** | | | | | | | | | |
| --- | --- | --- | --- | --- | --- | --- | --- | --- | --- |
| **On a typical WEEKDAY, how much time do you spend (from when you wake up until you go to bed) domingo the following?** | | | | | | | | | |
|  | **None** | **15 min. or less** | **30 min** | **1 hr** | **2 hrs** | **3 hrs** | **4 hrs** | **5 hrs** | **6 hrs or more** |
| 1. Watching television (including videos on VCR/DVD). |  |  |  |  |  |  |  |  |  |
| 2. Playing computer or video games. |  |  |  |  |  |  |  |  |  |
| 3. Sitting listening to music on the radio, tapes, or CDs. |  |  |  |  |  |  |  |  |  |
| 4. Sitting and talking on the pone. |  |  |  |  |  |  |  |  |  |
| 5. Doing paperwork or computer work (office work, emails, paying bills, etc.) |  |  |  |  |  |  |  |  |  |
| 6. Sitting reading a book or magazine. |  |  |  |  |  |  |  |  |  |
| 7. Playing a musical instrument. |  |  |  |  |  |  |  |  |  |
| 8. Doing artwork or crafts. |  |  |  |  |  |  |  |  |  |
| 9. Sitting and driving in a car, bus, or train. |  |  |  |  |  |  |  |  |  |
| **SEDENTARY BEHAVIOR: Weekend Day** | | | | | | | | | |
| **On a typical WEEKEEND DAY, how much time do you spend (from when you wake up until you go to bed) domingo the following?** | | | | | | | | | |
|  | **None** | **15 min. or less** | **30 min** | **1 hr** | **2 hrs** | **3 hrs** | **4 hrs** | **5 hrs** | **6 hrs or more** |
| 1. Watching television (including videos on VCR/DVD). |  |  |  |  |  |  |  |  |  |
| 2. Playing computer or video games. |  |  |  |  |  |  |  |  |  |
| 3. Sitting listening to music on the radio, tapes, or CDs. |  |  |  |  |  |  |  |  |  |
| 4. Sitting and talking on the pone. |  |  |  |  |  |  |  |  |  |
| 5. Doing paperwork or computer work (office work, emails, paying bills, etc.) |  |  |  |  |  |  |  |  |  |
| 6. Sitting reading a book or magazine. |  |  |  |  |  |  |  |  |  |
| 7. Playing a musical instrument. |  |  |  |  |  |  |  |  |  |
| 8. Doing artwork or crafts. |  |  |  |  |  |  |  |  |  |
| 9. Sitting and driving in a car, bus, or train. |  |  |  |  |  |  |  |  |  |
