# Supplementary material for: Quasi-experimental controlled study protocol to reduce sedentary lifestyle in patients with type 2 diabetes
Source: PLoS One. 2025 Sep 16;20(9):e0330393. doi: 10.1371/journal.pone.0330393 (PMC12440174; doi:10.1371/journal.pone.0330393)
Supplement: S5 Appendix — (DOCX) [file pone.0330393.s005.docx]

**APPENDIX 5. TRANSTHEORETICAL MODEL OF PHYSICAL EXERCISE CHANGE QUESTIONNAIRE (TMPECQ)**

DID NOT PRACTICE REGULAR PHYSICAL EXERCISE…

| 1 | Because I think I do not need it. | 1 2 3 4 5 |
| --- | --- | --- |
| 2 | Because I do not have access to a suitable place. | 1 2 3 4 5 |
| 3 | And I'm satisfied to be a sedentary person. | 1 2 3 4 5 |
| 4 | But I'm thinking that I should try to start a regular physical exercise program in the next 6 months. | 1 2 3 4 5 |
| 5 | But I would like to try some physical activity. | 1 2 3 4 5 |
| 6 | But I have already established a day and an hour to start regular physical exercise in the coming weeks. | 1 2 3 4 5 |
| 7 | And I do not plan to do it. | 1 2 3 4 5 |
| 8 | But I think it's worth doing. | 1 2 3 4 5 |
| 9 | But I already know where I'm going to practice physical exercise (gym, park, sports court ...). | 1 2 3 4 5 |
| 1  0 | Because I cannot count on my surroundings (family, friends ...). | 1 2 3 4 5 |
| 1  1 | But I wanted to start having a more active life. | 1 2 3 4 5 |
| 1  2 | Because I do not have energy (I do not feel like it). | 1 2 3 4 5 |
| 1  3 | But I have considered if I would be able to do it. | 1 2 3 4 5 |
| 1  4 | But I've been thinking that maybe I want to start. | 1 2 3 4 5 |
| 1  5 | But I consider it important. | 1 2 3 4 5 |
| 1  6 | But I've been thinking about the possibility of starting to do it. | 1 2 3 4 5 |
| 1  7 | And I do not worry. | 1 2 3 4 5 |
| 1  8 | But I am already preparing to start exercising in a group in the following weeks. | 1 2 3 4 5 |
| 1  9 | But I've already been with a friend to start exercising within the next few weeks. | 1 2 3 4 5 |
| 2  0 | But I've already been calling friends to find someone to start exercising with me in the next few weeks. | 1 2 3 4 5 |
| 2  1 | Because I do not consider it important. | 1 2 3 4 5 |
| 2  2 | Because I do not have time | 1 2 3 4 5 |

PERFORMING REGULAR PHYSICAL EXERCISE…

| 1 | I have been successful in exercising regularly and I plan to continue. | 1 2 3 4 5 |
| --- | --- | --- |
| 2 | I have recently started doing regular exercise. | 1 2 3 4 5 |
| 3 | I have been doing regular physical exercise for a long time and I intend to continue like this. | 1 2 3 4 5 |
| 4 | I have managed to keep doing physical exercise in the last six months. | 1 2 3 4 5 |
| 5 | I have started to do regular exercise and I plan to continue. | 1 2 3 4 5 |
| 6 | I have started to exercise regularly in the last six months. | 1 2 3 4 5 |
| 7 | Finally I exercise regularly. | 1 2 3 4 5 |
| 8 | I have been exercising for some time and I plan to continue. | 1 2 3 4 5 |
| 9 | I have completed more than six months doing regular physical exercise. | 1 2 3 4 5 |
