# Supplementary material for: Quasi-experimental controlled study protocol to reduce sedentary lifestyle in patients with type 2 diabetes
Source: PLoS One. 2025 Sep 16;20(9):e0330393. doi: 10.1371/journal.pone.0330393 (PMC12440174; doi:10.1371/journal.pone.0330393)
Supplement: S6 Appendix — (DOCX) [file pone.0330393.s006.docx]

**APPENDIX 6. INTERNATIONAL PHYSICAL ACTIVITY QUESTIONNAIRE (IPAQ)**

We are interested in finding out about the kinds of physical activities that people do as

part of their everyday lives. The questions will ask you about the time you spent being

physically active in the last 7 days. Please answer each question even if you do not

consider yourself to be an active person. Please think about the activities you do at

work, as part of your house and yard work, to get from place to place, and in your spare

time for recreation, exercise or sport.

Think about all the vigorous activities that you did in the last 7 days. Vigorous

physical activities refer to activities that take hard physical effort and make you breathe

much harder than normal. Think only about those physical activities that you did for at

least 10 minutes at a time.

1. During the last 7 days, on how many days did you do vigorous physical

activities like heavy lifting, digging, aerobics, or fast bicycling?

_____ days per week

No vigorous physical activities Skip to question 3

2. How much time did you usually spend doing vigorous physical activities on one

of those days?

_____ hours per day

_____ minutes per day

Don’t know/Not sure

Think about all the moderate activities that you did in the last 7 days. Moderate

activities refer to activities that take moderate physical effort and make you breathe

somewhat harder than normal. Think only about those physical activities that you did

for at least 10 minutes at a time.

3. During the last 7 days, on how many days did you do moderate physical

activities like carrying light loads, bicycling at a regular pace, or doubles tennis?

Do not include walking.

_____ days per week

No moderate physical activities Skip to question 5

4. How much time did you usually spend doing moderate physical activities on one

of those days?

_____ hours per day

_____ minutes per day

Don’t know/Not sure

Think about the time you spent walking in the last 7 days. This includes at work and at

home, walking to travel from place to place, and any other walking that you have done

solely for recreation, sport, exercise, or leisure.

5. During the last 7 days, on how many days did you walk for at least 10 minutes

at a time?

_____ days per week

No walking Skip to question 7

6. How much time did you usually spend walking on one of those days?

_____ hours per day

_____ minutes per day

Don’t know/Not sure

The last question is about the time you spent sitting on weekdays during the last 7

days. Include time spent at work, at home, while doing course work and during leisure

time. This may include time spent sitting at a desk, visiting friends, reading, or sitting or

lying down to watch television.

7. During the last 7 days, how much time did you spend sitting on a week day?

_____ hours per day

_____ minutes per day

Don’t know/Not sure

This is the end of the questionnaire, thank you for participating.
