# Supplementary material for: Quasi-experimental controlled study protocol to reduce sedentary lifestyle in patients with type 2 diabetes
Source: PLoS One. 2025 Sep 16;20(9):e0330393. doi: 10.1371/journal.pone.0330393 (PMC12440174; doi:10.1371/journal.pone.0330393)
Supplement: S7 Appendix — (DOCX) [file pone.0330393.s007.docx]

**APPENDIX 7. DIABETES QUALITY OF LIFE (DQoL)**

**Satisfaction**

**(1= very satisfied;2= moderately satisfied;3= ;4= moderately dissatisfied; 5= very dissatisfied)**

1. How satisfied are you with the amount of time it takes to manage your diabetes?

2. How satisfied are you with the amount of time you spend getting checkups?

3. How satisfied are you with the time it takes to determine your sugar level?

4. How satisfied are you with your current treatment?

5. How satisfied are you with the flexibility you have in your diet?

6. How satisfied are you with the burden your diabetes is placing on your family?

7. How satisfied are you with your knowledge about your diabetes?

8. How satisfied are you with your sleep?

9. How satisfied are you with your social relationships and friendships?

10. How satisfied are you with your sex life?

11. How satisfied are you with your work, school, and household activities?

12. How satisfied are you with the appearance of your body?

13. How satisfied are you with the time you spend ex ercising?

14. How satisfied are you with your leisure time?

15. How satisfied are you with life in general?

**Impact**

**(1= never;2= very seldom;3= sometimes;4= often;5= all the time)**

1. How often do you feel pain associated with the treatment for your diabetes?

2. How often are you embarrassed by having to deal with your diabetes in public?

3. How often do you have low blood sugar?

4. How often do you feel physically ill?

5. How often does your diabetes interfere with your family life?

6. How often do you have a bad night's sleep?

7. How often do you find your diabetes limiting your social relationships and friendships?

8. How often do you feel good about yourself?

9. How often do you feel restricted by your diet?

10. How often does your diabetes interfere with your sex life?

11. How often does your diabetes keep you from driving a car or using a machine (e.g., a typewriter)?

12. How often does your diabetes interfere with your exercising?

13. How often do you miss work, school, or household duties because of your diabetes?

14. How often do you find yourself explaining what it means to have diabetes?

15. How often do you find that your diabetes interrupts your leisure-time activities?

16. How often do you tell others about your diabetes?

17. How often are you teased because you have diabetes?

18. How often do you feel that because of your diabetes you go to the bathroom more than others?

19. How often do you find that you eat something you shouldn't rather than tell someone that you have diabetes?

20. How often do you hide from others the fact that you are having an insulin reaction?

**Worry: Social/Vocational**

**(0= does not apply;1= never;2= very seldom;3= sometimes;4= often;5= all the time)**

1. How often do you worry about whether you will get married?

2. How often do you worry about whether you will have children?

3. How often do you worry about whether you will not get a job you want?

4. How often do you worry about whether you will be denied insurance?

5. How often do you worry about whether you will be able to complete your education?

6. How often do you worry about whether you will miss work?

7. How often do you worry about whether you will be able to take a vacation or a trip?

**Worry: Diabetes Related**

**(0= does not apply;1= never;2= very seldom;3= sometimes;4= often;5= all the time)**

1. How often do you worry about whether you will pass out?

2. How often do you worry that your body looks different because you have diabetes?

3. How often do you worry that you will get complications from your diabetes?

4. How often do you worry about whether someone will not go out with you because you have diabetes?
