# Supplementary material for: Quasi-experimental controlled study protocol to reduce sedentary lifestyle in patients with type 2 diabetes
Source: PLoS One. 2025 Sep 16;20(9):e0330393. doi: 10.1371/journal.pone.0330393 (PMC12440174; doi:10.1371/journal.pone.0330393)
Supplement: Attachment — Submitted filename: Response to Reviewers.docx [file pone.0330393.s009.docx]

Raquel Sainz, PhD

Editor-in-Chief

*PLOS One*

Logroño, June 21, 2025

Dear Editor-in-Chief,

We sincerely thank the Editorial Board for their consideration in our manuscript and the reviewers for their insightful and constructive feedback. We have addressed all the reviewers' suggestions to improve the manuscript and have implemented revisions throughout.

We are truly grateful for the feedback received, which has significantly contributed to refining the clarity, methodological rigor, and overall quality of the manuscript.

We are pleased to submit this improved version of the manuscript for your consideration for publication in *PLOS ONE*. Alongside the revised manuscript, we are including a document that highlights all modifications, as well as a detailed response to each of the reviewers' comments explaining how we addressed their feedback as follows.

Thank you for your time and consideration.

Sincerely,

On behalf of all the authors,

Raquel Sainz

University of La Rioja

Logroño, La Rioja

**Response to Referee #1**

We sincerely appreciate the time devoted to reviewing our manuscript and the valuable comments provided, which have helped us to clarify important aspects of the study. We have carefully addressed each of the points raised and made the corresponding changes to the text, which are described below.

(1) **“Half will be allocated to the control group…” Please clarify how participants will be assigned to different groups.**

We have clarified in the manuscript that the assignment of participants to the groups will be carried out sequentially and alternately, based on the order in which the informed consent is signed. That is, the first participant to sign the consent form will be assigned to the intervention group, the second to the control group, and so on. In addition, we have specified the number of participants in each group: 85 in the intervention group and 84 in the control group. **(Lines 126-129)**

(2) **The IG group will be divided into two groups based on age. Please be more specific. Also how will the age cutpoint be selected? Also, will there be age/group-specific effect or heterogeneity?**

The cut-off point at 65 years of age was established solely for organizational and logistical reasons, as this age is a commonly used threshold to distinguish between the adult and older adult populations in the healthcare field. This division facilitates the formation of more generationally homogeneous groups and allows for better management and comfort during group sessions, without implying any differences in the intervention’s content or focus. **(Lines 182-188)**

(3) **Please comment on the generalizability of a single-center study as proposed in this study.**

We appreciate your comment regarding the generalizability of the study. While we acknowledge that this design may entail certain limitations concerning external validity, we consider that in our case this limitation is mitigated by the fact that the sample includes a large proportion of the type 2 diabetes community in the La Rioja region, through the ASDIR. This extensive coverage within a single regional community provides considerable sociodemographic diversity, thereby enhancing the representativeness of the sample and strengthening the applicability of the findings to similar populations. Moreover, the components of the intervention (in-person health education and telephone follow-up) are easily reproducible and do not require complex resources, which facilitates their implementation in other similar settings. **(Lines 491-496)**

**Response to Referee #2**

We sincerely appreciate your kind words regarding the manuscript and the recognition of the proposed intervention, as well as your acknowledgment of the potential impact this protocol may have on public health improvement for patients with type 2 diabetes. We deeply value your critical and constructive observations, which have been instrumental in enhancing the quality and clarity of the manuscript. We have carefully reviewed each of the points raised and made the necessary revisions to strengthen the transparency and reproducibility of the study. Below, we detail how we have addressed each of the specific comments.

SPECIFIC POINTS

**Introduction**

(1) **Clearly specify the global and local prevalence of sedentary behavior in type 2 diabetes patients to highlight the relevance of the study in the chosen setting.**

The following information has been added in **lines 56–61**: In Spain, a study conducted by the Spanish Diabetes Society (SED) and the Spanish Diabetes Federation (FEDE) identified that approximately four out of ten people with diabetes lead a sedentary lifestyle (1).
At the local level, nearly one-quarter of the population in La Rioja exhibits high levels of sedentary behavior, with women showing the highest prevalence (2).
Although specific local data disaggregated by chronic diseases are not available, these figures highlight the need to develop local studies that allow for the design of interventions tailored to the characteristics and needs of this population.

(2) **Explicitly mention the novelty of your intervention: e.g., the combined use of face-to-face group sessions, individual consultations, and regular telephone follow-ups specifically delivered by nurses.**

We have added an explanation in the introduction highlighting the novelty of the intervention (**lines 91–96**). This novelty lies in the combination of educational and motivational components, along with both telephone and in-person follow-up, all conducted by nursing staff. This comprehensive design, based on educational and motivational tools, facilitates personalized, sustainable behavior change that is potentially applicable to real-world healthcare settings.

**Methods**

(1) **Clarify the rationale behind choosing a 5% margin of error and anticipated 15% dropout rate; consider referencing similar studies or standard guidelines.**

A 95% confidence level and a 5% margin of error were used—parameters widely accepted in epidemiological and community intervention studies as the standard to ensure precise estimation with a reasonable probability of error (3). This choice allows for a balance between statistical accuracy and the practical feasibility of the study.

Furthermore, an anticipated attrition rate of 15% was considered based on similar studies involving adult populations with chronic conditions such as type 2 diabetes, where dropout rates range from 10% to 20% during interventions lasting several months (4–7). This estimate takes into account factors such as advanced age, disease burden, and potential low adherence to behavior change programs. **(Lines 119-125)**

(2) **Provide more details regarding randomization procedures; clarify if allocation concealment techniques (e.g., sealed envelopes, computer-generated sequences) are used to avoid selection bias.**

We have clarified in the manuscript that participant allocation will be conducted using a systematic alternate method (1:1) according to the order of inclusion, without concealment techniques, and therefore it is not a strict randomization. Additionally, it has been noted that the principal investigator will be responsible for both data collection and analysis, so assessor blinding will not be applied. These limitations have been taken into account in the study design and will be addressed in the discussion of the results. **(Lines 164-170)**

**Eligibility criteria**

(1) **Define clearly what constitutes “minimal physical fitness” as an inclusion criterion; specify measurable criteria or validated instruments.**

Thank you for your valuable observation. In response to your comment, we have clarified in the manuscript the definition of the concept of "minimum physical fitness" as an inclusion criterion. Specifically, it is defined as the functional ability to rise from and sit down in a chair unassisted, walk short distances (at least 100 meters) independently, and perform light household tasks without difficulty (e.g., making the bed, washing dishes, or preparing simple meals). This fitness is assessed through clinical observation and participant self-report during the initial interview, which ensures that participants have the minimum physical capacity required to safely take part in the intervention. **(Lines 144-149)**

(2) **Consider explaining why certain exclusion criteria (recent surgery, oncological treatment, cognitive impairment) were selected and how these might impact study outcomes.**

We have included a more precise explanation regarding these exclusion criteria in **lines 150-158** of the manuscript.

Recent surgery

Regarding the criterion of recent surgical interventions that limit mobility, it has been refined to improve its accuracy and applicability, focusing on recent major surgical procedures that result in functional limitations or that, based on clinical judgment, have not allowed sufficient recovery to participate in the study.

Functional recovery after surgery largely depends on the type of procedure, the patient’s baseline condition, and the presence of comorbidities. Furthermore, the restoration of strength, mobility, and participation in daily or recreational activities may be compromised for weeks or even months depending on the complexity of the surgery and potential postoperative complications.

Since the intervention proposed in this study requires a minimum level of mobility for proper implementation and to ensure participant safety, patients who have undergone recent major surgeries are excluded. Major surgery is defined as procedures requiring general or regional anesthesia that may compromise functional mobility for a prolonged period (e.g., hip or knee arthroplasty, spinal surgery, major abdominal or thoracic surgery, among others). Patients with minor or rapidly recovering surgeries (such as uncomplicated appendectomy or laparoscopic cholecystectomy) are not unnecessarily excluded, provided they have regained their usual functional mobility and present no limitations that interfere with participation in the intervention. This measure also aims to ensure sample homogeneity and the validity of the results obtained.

Patients undergoing active oncological treatment

Chemotherapy is a common component of treatment for many oncological diseases at some stage of their course. Unfortunately, antineoplastic drugs can cause side effects that negatively impact patients’ quality of life. These effects may occur immediately (hours or days), early (days or weeks), delayed (weeks or months), or late (months or years). Among them, asthenia — a persistent feeling of physical, emotional, and mental exhaustion — is one of the most prevalent complications, affecting up to 90% of patients during treatment and persisting in more than 50% after its completion (8). Other frequent symptoms include pain, psychological disturbances, sleep disorders, gastrointestinal issues, anorexia, and toxicity (9). These symptoms, resulting from both the disease and its treatment, could limit patients’ active participation in the intervention and compromise the validity of the outcomes obtained.

Cognitive impairment

Cognitive impairment may represent a prodromal stage of Alzheimer’s disease, especially when manifested as mild cognitive impairment. Although significant impact on activities of daily living may not be observed in many cases, evidence shows that this condition is associated with difficulties in acquiring verbal and non-verbal information, language skill impairments, attention problems, executive dysfunction, and reduced cognitive flexibility — cognitive symptoms that can appear from early stages (10–12).

For this reason, cognitive impairment has been considered an exclusion criterion, as it may hinder understanding and adherence to the intervention and could interfere with the reliability of the responses provided in the questionnaires used.

**Interventions and activities**

(1) **Specify the frequency, duration, and detailed content of face-to-face group sessions and individual visits to enhance reproducibility.**

The requested details have been incorporated into the manuscript and can be found in **lines 189-215 and 222-228**. These sections precisely describe the frequency, content, and approximate duration of the in-person group sessions and individual visits. Additionally, detailed information regarding the purpose of the telephone calls has been included.

(2) **Clarify who will conduct motivational interviewing by phone, and provide their training background or qualifications to ensure intervention consistency.**

The requested information has been added to the manuscript (see **lines 216-221**). The motivational telephone interviews will be conducted by a nurse with a master’s degree in biomedical research and additional training in diabetes and motivational communication, together with a diabetes nurse educator with extensive experience and specialized training in this disease. Both professionals have been trained in the protocol and interview script to ensure the consistency and quality of the intervention.

**Outcome measurements**

(1) **Sedentary behavior measurement:**
    1.1. **Clearly define the thresholds for classifying sedentary versus active behaviors from accelerometer data.**

The following information has been added to the manuscript, in **lines 324-332**. Sedentary behavior will be defined as any period with ≤100 counts per minute (cpm) recorded by the ActiTrainer accelerometer. This threshold has been widely accepted in the scientific literature and specifically validated in adults against reference systems such as the IDEEA monitor, considered an almost gold standard for postural measurement, showing good agreement in estimated sedentary time (13). Additionally, the cut-off points established by Freedson et al. (1998) will be used to classify light physical activity (101–1951 cpm), moderate (1952–5724 cpm), vigorous (5725–9498 cpm), and very vigorous (>9498 cpm), according to the estimated energy expenditure in METs (14).

1.2. **Specify how missing accelerometer data (e.g., due to non-compliance or technical issues) will be handled.**

Thank you for your observation. For data processing, only days with a minimum of 10 hours of device wear time during waking hours will be considered valid. At least 4 valid days (including at least one weekend day) will be required for the recording to be deemed representative, following criteria established in previous studies (13–15). Periods of non-wear, due to non-compliance or technical issues, will be excluded from the analysis using validated algorithms for non-wear detection, which automatically identify periods without use based on consecutive intervals of no movement recordings longer than 60 minutes (16). If any participant does not meet the minimum valid days criterion, a sensitivity analysis will be conducted and the number of valid days per participant will be reported to ensure transparency and data quality. This information has been incorporated into the manuscript, in **lines 333-342**.

(2) **Motivation to change:**

**Briefly clarify the clinical interpretation of scores from the TMPEC questionnaire—what specific score ranges indicate meaningful shifts in motivational stages.**

As stated in the manuscript, the interpretation of the TMPEC questionnaire results is carried out by comparing the mean scores obtained in each subscale corresponding to a stage of change. Although the questionnaire is validated in Spanish (17), there is no exact numerical threshold established to define significant changes. However, from a clinical perspective, an increase of 0.5 points or more in the mean score of a subscale may be considered indicative of a significant change in motivation. Additionally, a shift in the highest score from one stage to another (for example, from “Contemplation” to “Preparation”) suggests progression in the motivational process. Mean scores above 4 reflect a strong identification with that stage. **(Lines 354–362)**

(3) **Clinical parameters:**

**Specify if blood parameters will be collected under fasting conditions and detail the handling and analysis methods (e.g., laboratory accreditation, biochemical assay types).**

Blood parameters will always be collected under fasting conditions, after a minimum period of 8 hours without intake of food or beverages. The samples will be processed and analyzed in a laboratory accredited according to the current regulations of the Autonomous Community of La Rioja. Specifically, the reference laboratory of the Autonomous Community of La Rioja is located at the Hospital Universitario San Pedro de Logroño, which holds accreditation from the National Accreditation Entity (ENAC) in accordance with the UNE-EN ISO 15189:2023 standard for its Clinical Analysis Service. This accreditation covers both the central laboratory and the microbiology laboratory, endorsing the laboratory’s technical competence to perform the accredited tests and ensuring the quality and traceability of the results. The handling and transport of the samples will be carried out following standardized quality protocols to guarantee their stability and analytical reliability. Biochemical analyses will be performed using automated techniques, employing equipment calibrated and periodically validated according to clinical laboratory standards. **(Lines 286-291)**

**Statistical Analysis**

(1) **Clearly justify the selection of repeated measures ANOVA or Friedman’s test; specify conditions under which each test will be applied (normality checks, variance homogeneity tests).**

We appreciate the comment regarding the justification for using repeated measures ANOVA and the Friedman test. The following clarification will be incorporated in **lines 398-408** of the manuscript:

Repeated measures analysis of variance (ANOVA) will be used to compare differences in continuous variables over time between groups when the necessary statistical assumptions are met. Specifically, residual normality will be assessed using the Shapiro-Wilk test, and homogeneity of variances will be verified with Mauchly’s test for sphericity. If sphericity is met, the standard ANOVA will be applied; otherwise, Greenhouse-Geisser or Huynh-Feldt corrections will be used to adjust the degrees of freedom.

In cases where variables do not meet the normality assumption, or when data are ordinal or clearly non-parametric in distribution, the non-parametric Friedman test will be used to analyze differences between repeated measurements. This test allows comparison of changes over time without assuming normal distribution or homogeneity of variances.

Thus, the choice between repeated measures ANOVA and the Friedman test will strictly depend on whether these assumptions are met or violated, ensuring the validity of the statistical results.

(2) **Explain your approach to handling missing data clearly; state whether techniques like intention-to-treat analysis, multiple imputation, or sensitivity analyses will be used.**

In this study, a rigorous approach will be adopted to handle missing data in order to minimize bias and preserve the validity of the analysis.
Initially, a descriptive analysis will be performed to identify the pattern and proportion of missing data. Depending on the nature and amount of missing data, an intention-to-treat (ITT) analysis will be conducted, including all participants assigned to groups regardless of adherence or losses during follow-up.
For the specific handling of missing data in quantitative variables, multiple imputation using the multiple imputation by chained equations (MICE) method will be applied. This technique generates several complete datasets by imputing plausible values based on relationships among observed variables, improving precision and reducing bias in estimates.
Additionally, a sensitivity analysis will be performed comparing the results obtained with imputed data versus those with complete-case data (per-protocol analysis), to assess the robustness of conclusions under different missing data handling scenarios.
This approach ensures proper management of missing data and strengthens the internal validity of the study. **(Lines 413-420)**

(3) **Clarify if subgroup analyses are pre-specified or exploratory, and specify how multiple testing (e.g., Bonferroni or Benjamini-Hochberg corrections) will be handled to minimize type I errors.**

We appreciate the comment regarding subgroup analyses. In our protocol, subgroup analyses are considered primarily exploratory and will be conducted to identify potential differences in response to the intervention according to relevant participant characteristics such as age, sex, and level of motivation to change. These analyses are not part of the predefined primary or secondary objectives; therefore, the results will be interpreted with caution.

To minimize the risk of type I errors associated with multiple comparisons, corrections for multiple testing will be applied, prioritizing the Benjamini-Hochberg method to control the false discovery rate (FDR), given its greater power compared to more conservative methods such as Bonferroni. In cases where a small number of comparisons are performed, the Bonferroni correction will also be considered.
This approach ensures an appropriate balance between detecting relevant effects and controlling for false positive results. **(Lines 427-433)**

**Ethical Considerations**

(1) **Confirm the planned frequency of ethical audits and how protocol amendments will be communicated to the ethics committee and participants.**

The information has been added in **lines 254-259**.
Ethical audits are planned to be conducted annually and at the conclusion of the study. The research team commits to informing the ethics committee of any substantial protocol modifications, such as changes in inclusion/exclusion criteria, data collection instruments, or the intervention design. Such amendments will be formally communicated to the committee through a written report, sent via email along with the necessary supplementary documentation, and will be submitted for evaluation and approval prior to implementation.

If relevant changes affecting participants occur (for example, regarding procedures, study duration, or session content), these will be communicated directly to the participants via an informative letter or in person, requesting renewed informed consent if necessary.

**Discussion**

(1) **Explicitly acknowledge potential limitations, including generalizability due to single-center design, the risk of contamination between groups, and the possible impact of seasonal variation on sedentary behavior.**

This study presents some limitations that should be considered and have been incorporated in **lines 491-505**.

Firstly, the single-center design may limit the generalizability of the results to other populations or healthcare settings. However, one of the strengths of the study is that, despite its single-center nature, the sample includes a broad and diverse community of individuals with type 2 diabetes from across the La Rioja region, which provides sociodemographic variability and strengthens the external validity of the findings within similar populations.

Since participants in the control and intervention groups may share community or healthcare environments, there is a potential risk of contamination between groups, which could reduce the actual difference in observed effects. Another limitation to consider is the lack of blinding for both participants and researchers, which may affect the objectivity of the results. Although this phenomenon is common in behavioral studies, it must be taken into account when interpreting the findings.

Furthermore, the study covers a 12-month intervention and follow-up period, so there is a possibility that seasonal variation may influence sedentary behavior and physical activity (for example, lower activity during winter), this factor should be considered when interpreting the results.

(2) **Recommend prospective follow-up beyond the 12-month period to evaluate the long-term sustainability of behavior changes and clinical outcomes.**

Thank you for your recommendation. Although this study includes a 12-month measurement without intervention during the second semester, allowing for the observation of maintenance of change after the completion of the active intervention, it is recommended that future studies consider a prospective follow-up beyond 12 months. This would allow for a more precise evaluation of the sustainability of behavioral changes and their impact on long-term clinical outcomes in people with type 2 diabetes. (**Lines 506-511**)

(3) **Discuss possible economic implications or cost-effectiveness analysis for implementing such nurse-led interventions at scale within health systems.**

The proposed intervention, led by nursing professionals, presents a potentially cost-effective profile for the healthcare system. It focuses on education, the promotion of healthy habits, and behavioral support—strategies that require a relatively low initial investment compared to other clinical or pharmacological interventions.

Regarding large-scale implementation, cost-effectiveness analysis becomes highly important, as the expansion of the intervention must ensure an optimal balance between economic resources employed and the health benefits obtained in terms of public health. Firstly, the scalability of the intervention, favored by its replicability from nursing practice, allows the marginal costs associated with its expansion to be proportional and not compromise the financial sustainability of the system. However, adaptation to different contexts or population sizes may require an additional initial investment aimed at professional training, team coordination, and logistical adjustments—key aspects to ensure proper adaptation and execution. Once these initial challenges are overcome, the intervention is expected to generate substantial savings in the medium and long term, mainly derived from the reduction of chronic complications, decreased hospitalizations, and improved disease control, which translates into a lower care burden for health services, optimizing public resources and allowing their reallocation to areas with greater demand.

Moreover, the intervention contributes to improving the quality of care and fostering patient autonomy in disease management. Beyond generating new knowledge about community interventions aimed at reducing sedentary behavior in people with chronic diseases, this project pursues clear practical application and transfer to the healthcare system of La Rioja, promoting innovation in nursing care, evidence-based decision making, and the sustainability of the system in the medium and long term.

In the upcoming phases of the project, an economic analysis is planned through the systematic collection of data on implementation costs and the monitoring of clinical and quality-of-life indicators, in order to model the economic impact of the intervention. **(Lines 512-529)**

**REFERENCES**

1. Rojo-Martínez G, Valdés S, Soriguer F, Vendrell J, Urrutia I, Pérez V, et al. Incidence of diabetes mellitus in Spain as results of the nation-wide cohort di@bet.es study. Sci Rep. 2020 Dec 1;10(1).
2. Ministerio de Sanidad, Consumo y Bienestar Social [Internet]. Madrid: Gobierno de España;2024 [cited June 19, 2025]. Available from: https://www.sanidad.gob.es/

3.	Hulley SB, Cummings SR, Browner WS, Grady DG, Newman TB. Designing Clinical Research. 3rd edition. Philadelphia: Lippincott Williams & Wilkins; 2013.

4.	Márquez Contreras E, Martell Claros N, Gil Guillén V, Casado Martínez JJ, Martín De Pablos JL, Ferraro García J, et al. [Therapeutic adherence to insulin in the treatment of type 2 diabetes mellitus: the CUMINDIAB study]. Aten Primaria. 2012 Feb;44(2):74–81. Spanish.

5.	Cabré C, Colungo C, Vinagre I, Jansà M, Conget I. A therapeutic education program with a diabetes specialist nurse for type 2 diabetes patients using insulin in a primary care setting. A diabetes education program with a diabetes specialist nurse in a primary care setting. Endocrinol Diabetes Nutr. 2021;68(10):628–635. 22.

6.	Plotnikoff RC, Costigan SA, Karunamuni ND, Lubans DR. Community-based physical activity interventions for treatment of type 2 diabetes: A systematic review with meta-analysis. Front Endocrinol (Lausanne). 2013 Jan 29;4:3.

7.	Pi-Sunyer X. The Look AHEAD Trial: A Review and Discussion of Its Outcomes. Vol. 3, Current Nutrition Reports. Current Science Inc.; 2014. p. 387–91.

8.	Gloria Ariño Gadea, Miriam Brocate San Juan, Raquel Moreno Fraile, Ana Ruata Laclaustra, María Blanca Vera, Mar Aquilué Berdún. [Advice on side effects in patients undergoing chemotherapy treatment]. Rev Sanitaria Investig. 2023;4(3). Spanish.

9.	Ferreiro J, García JL, Barceló R, Rubio I. [Adverse effects of chemotherapy treatment]. Gac Med Bilbao. 2003;100:69–74. Spanish.

10.	Tsantali E, Economidis D, Tsolaki M. Could language deficits really differentiate Mild Cognitive Impairment (MCI) from mild Alzheimer’s disease? Arch Gerontol Geriatr. 2013 Nov;57(3):263–70.

11.	Reinvang I, Grambaite R, Espeseth T. Executive dysfunction in MCI: Subtype or early symptom. International Journal of Alzheimer’s Disease. 2012.

12.	Malek-Ahmadi M, Davis K, Belden CM, Jacobson S, Sabbagh MN. Informant-reported cognitive symptoms that predict amnestic mild cognitive impairment. BMC Geriatr. 2012 Feb 3;12:3.

13.	Matthews CE, Chen KY, Freedson PS, Buchowski MS, Beech BM, Pate RR, et al. Amount of time spent in sedentary behaviors in the United States, 2003-2004. Am J Epidemiol. 2008 Apr;167(7):875–81.

14.	Freedson PS, Melanson E, Sirard J. Calibration of the Computer Science and Applications, Inc. accelerometer. Med Sci Sports Exerc. 1998 May;30(5):777–81.

15.	Troiano RP, Berrigan D, Dodd KW, Masse LC, Tilert T, McDowell M. Physical activity in the United States measured by accelerometer. Med Sci Sports Exerc. 2008 Jan;40(1):181–8.

16.	Choi L, Liu Z, Matthews CE, Buchowski MS. Validation of accelerometer wear and nonwear time classification algorithm. Med Sci Sports Exerc. 2011 Feb;43(2):357–64.

17.	Leyton M, Batista M, Lobato S, Jiménez R. [Validation of the questionnaire of the Transtheoretical Model of Physical Exercise Change]. Rev Int Med Cienc Act Fís Deporte. 2019;19(74):329–50. Spanish.
