# Supplementary material for: Quasi-experimental controlled study protocol to reduce sedentary lifestyle in patients with type 2 diabetes
Source: PLoS One. 2025 Sep 16;20(9):e0330393. doi: 10.1371/journal.pone.0330393 (PMC12440174; doi:10.1371/journal.pone.0330393)
Supplement: Attachment — Submitted filename: Response_to_reviewers_auresp_2.docx [file pone.0330393.s010.docx]

Raquel Sainz, PhD

Editor-in-Chief

*PLOS One*

Logroño, July 15, 2025

Dear Editor-in-Chief,

We appreciate the opportunity to revise our manuscript and would like to sincerely thank the Editorial Board for their continued consideration. We also wish to thank the reviewer for the valuable comments that have helped us further refine our work.

In response to the suggestions provided, we have made the corresponding modifications to improve the manuscript. In the following pages, we detail our point-by-point responses, explaining the changes made and how each comment has been addressed.

We are pleased to resubmit the revised version of our manuscript for your consideration for publication in *PLOS ONE*, with the hope that the improvements introduced meet the expectations of the journal.

Thank you for your time and kind consideration.

Sincerely,

On behalf of all the authors,

Raquel Sainz

University of La Rioja

Logroño, La Rioja

**Response to Referee #2**

We are grateful for your thorough review and thoughtful suggestions. We have carefully considered each of your comments and implemented the necessary revisions, as detailed below.

(1) **You added national figures (Spain 40 %, La Rioja ≈ 25 %), but a global estimate is still missing. Please insert a concise line with a reliable worldwide figure for sedentary behaviour in adults with T2DM—for example, “≈ 60 % of adults with type 2 diabetes sit ≥ 8 h/day” (cite an authoritative source).**

Updated data regarding sedentary behavior in adults with type 2 diabetes mellitus worldwide have been incorporated into the manuscript **(Lines 57-60)**. Specifically, the results of a systematic review and meta-analysis published in 2024, which included 12 studies conducted in various countries with a total sample of 1,446,076 individuals, estimated that the prevalence of prolonged sedentary behavior among patients with type 2 diabetes is 52.3%, with higher rates in women than in men (60.3% vs. 56.5%) (1). The Maastricht Study reported that patients with type 2 diabetes spend an average of 10.1 hours per day engaged in sedentary behaviors (2). These findings are consistent with other recent studies, which also indicate high levels of sedentary behavior, with daily averages close to 10 hours (3).

(2) **Allocation is now sequential and non-concealed, which is appropriate only for a quasi-experimental design. Yet the manuscript alternates between “quasi-experimental” and “clinical trial.”
    • Option A: adopt true randomisation (computer-generated sequence + sealed opaque envelopes), or
    • Option B: keep sequential allocation, label the study consistently as a “quasi-experimental controlled study,” and discuss the resulting selection-bias risk in Methods and Limitations.**

On Friday, March 7, we received a message from the editorial staff stating the following:

“*The in-house editorial staff feels that your study meets the World Health Organization definition of a clinical trial because it is a prospective study in which participants were assigned to 'Nurse directed behavioral intervention' to investigate the effects on 'Anthropometric measurements/ Blood pressure and blood parameters'. Please change your manuscript’s article type to ‘Clinical Trial’ when you resubmit your manuscript*.”

Additionally, we were requested to submit the CONSORT statement despite already having uploaded the TREND checklist to the submission platform. This caused some confusion, as the TREND format is more appropriate for quasi-experimental studies.

However, we would like to clarify that the study has consistently followed a non-randomized design, with sequential and non-concealed allocation. Therefore, we have decided to maintain this allocation method and, accordingly, to consistently label the study as a “quasi-experimental controlled study” throughout the manuscript.

Information regarding this aspect has been incorporated into **lines 166–170** of the Methods section. Furthermore, a discussion of the potential risk of selection bias inherent to this design has been included in **lines 501–510** of the Limitations section. We acknowledge that the lack of full randomization may impact internal validity; therefore, we have detailed the strategies implemented to minimize this bias and contextualized how this affects the interpretation of the results.

In line with this correction, we have updated the study title, removed the CONSORT checklist, and uploaded the TREND checklist, which better suits the methodological characteristics of our study.

Additionally, since the study is registered at ClinicalTrials.gov, the information has also been updated in the submission platform, and the version of the manuscript includes the registration reference.

We sincerely apologize for any confusion this may have caused and thank you for your understanding and guidance.

(3) **Please state how multiplicity will be controlled for the full set of primary and secondary outcomes (e.g., Bonferroni, Benjamini-Hochberg) or justify why no adjustment is necessary**.

We appreciate the comment regarding the control of error due to multiple testing. After careful consideration, we have chosen to apply the Bonferroni correction to adjust the significance level in the variable analyses, in order to minimize the risk of false positives and ensure that the results are reliable and robust. Given that the number of comparisons performed is relatively small, we consider this conservative correction to be feasible without excessively compromising statistical power. Furthermore, we believe that this strategy is appropriate in our clinical context, where the accuracy and reliability of the results are essential to guide future recommendations for the intervention of patients with type 2 diabetes **(Lines 433-438)**.

**REFERENCES**

1. Salari N, Ahmadi M, Ghasemi H, Yarani R, Mohammadi M. The global prevalence of sedentary time in diabetes and metabolic syndrome: a systematic review and meta-analysis. Iran J Public Health. 2024 Sep;53(9):2020–9. <https://doi.org/10.18502/ijph.v53i9.16455>
2. Van der Berg JD, Stehouwer CD, Bosma H, Van der Velde JH, Willems PJ, Savelberg HH, et al. Associations of total amount and patterns of sedentary behaviour with type 2 diabetes and the metabolic syndrome: The Maastricht Study. Diabetologia. 2016 Apr;59(4):709-18. <https://doi.org/10.1007/s00125-015-3861-8>
3. Zhang X, Yang D, Luo J, Wang Y, Zhang Y, Li X, et al. Determinants of sedentary behavior in community-dwelling older adults with type 2 diabetes based on the behavioral change wheel: a path analysis. *BMC Geriatr*. 2024;24:502. <https://doi.org/10.1186/s12877-024-05076-0>
